# Children’s participation in school: a cross-sectional study of the relationship between school environments, participation and health and well-being outcomes

**DOI:** 10.1186/1471-2458-14-964

**Published:** 2014-09-17

**Authors:** Yetunde O John-Akinola, Saoirse Nic-Gabhainn

**Affiliations:** Health Promotion Research Centre, School of Health Sciences, College of Medicine, Nursing and Health Sciences, National University of Ireland, Galway, Ireland

**Keywords:** Children’s participation, School socio-ecological environment, Health and well-being outcomes, Health promoting schools, Ireland

## Abstract

**Background:**

Schools are a key setting for health promotion and improvement activities and the psycho-social environment of the school is an important dimension for promoting the health and well-being of children. The development of Health Promoting Schools (HPS) draws on the settings-based approach to health promotion and includes child participation as one of its basic values. This paper investigates the relationships between child participation, the school environment and child outcomes.

**Methods:**

Study participants were recruited from nine primary schools, three of which were designated as Health Promoting Schools (HPS). Each HPS was matched with two non-HPS (NHPS) with similar characteristics. Two hundred and thirty-one pupils in the 4th-6th class groups completed self-report questionnaires to document their perspectives on the school socio-ecological environment, how they take part in school life, school processes and their health and well-being.

**Results:**

School participation was measured with four scales: participation in school decisions and rules, school activities, school events and positive perception of school participation. The differences in the reported mean score for three of the four scales were marginal and not statistically significant. However, the mean score for reported positive perception of school participation was significantly lower (χ^2^ = 5.13, df =1, p < 0.05) among pupils in HPS (mean = 26.03; SD 3.37) compared to NHPS (mean = 26.30; SD 3.36). Participation in school decisions and rules (OR 1.22, 95% CI 1.12-1.33), participating in school activities (OR 1.20, 95% CI 1.10-1.31), participating in school events (OR 1.19, 95% CI 1.10-1.29) and reported positive perception of school participation (OR 1.26, 95% CI 1.15-1.39) were all positively associated with health and well-being outcomes for all pupils. Logistic regression analyses indicated positive associations between school participation and school socio-ecological environment.

**Conclusions:**

These findings suggest that school participation is important for children in schools and is relevant for improved school environment, relationships and positive health and well-being outcomes. The positive associations between school participation and school socio-ecological environment and health and well-being outcomes suggests that pupil health and well-being and school relationships could be improved or sustained by providing or supporting an environment that encourages pupil participation in school life.

**Electronic supplementary material:**

The online version of this article (doi:10.1186/1471-2458-14-964) contains supplementary material, which is available to authorized users.

## Background

The ecological perspective on health promotion describes behaviour as being affected by the relationships or interconnections between different levels of influence within the environment [1]. Ecological models can be adapted to investigate the effects of the setting in which an individual functions and their ability to make health promoting choices. The theoretical background to this is that behaviour does not take place in a vacuum. As Stokols [2] argued, there are mutual influences between individuals and their environments, each effecting the other, both as individuals and in groups. In the Health Promoting Schools (HPS) movement, health is considered to be holistic and to be generated from both social and ecological processes. The HPS encourages practices that improve the health and well-being of pupils and the whole school community [3] and emphasises the importance of the school setting and environment in contributing to the development of children and young people’s health-related competencies and lifestyles [4].

The HPS model includes the school environment and seeks to provide a supportive environment for pupils in order to create an atmosphere that encourages holistic learning and development. The core goal of HPS is the empowerment of the whole school environment, both for staff and students and at a collective level [5, 6]. Children’s participation in school life, for the purposes of promoting empowerment, represents one of the key pillars and strategies of the HPS model [3, 6]. It has been argued that the close connection or association of children with their environment, such as in schools, has the potential to constitute a strong determinant for the enhancement of children’s participation in the school setting [5]. Thus, from a HPS perspective, the structure or framework of the school environment - the policies, management structure, ‘feel’ of the school in terms of the social environment, the physical environment, school ethos and curriculum - are hypothesised to be linked with pupil participation in the life of the school. This study sought to examine the relationships between participation of pupils and the socio-ecological environment of schools.

Research has suggested that child participation in school, which represents one of the key values of HPS, has the potential to foster the development of pupils’ self-confidence and self-esteem [7, 8] and its impact on students’ positive views of their school has been highlighted [9]. Child participation has been associated with positive health and well-being of pupils [9] and could be beneficial in enhancing positive health outcomes [8]. However, much of the focus on participation has been on the processes involved in engaging young people in interventions specifically designed to improve aspects of their health and well-being, usually focusing on health related behaviours e.g., [7]. The perspective in this study, in contrast, is that the general participation of pupils in everyday school life, not just in specific projects, could be related to their general well-being. If demonstrated, this may imply that health and well-being could be improved or sustained by efforts to engage with and listen to pupils during the normal school day.

This study also sought to document the perceptions of primary school pupils in Ireland on participation in school, and to determine the extent to which children’s participation in school life was associated with the reported health and well-being of children.

The study hypothesis is that school participation is associated with the school socio-ecological environment and with pupils’ health and well-being outcomes.

## Methods

### Introduction

The research design used for this study was mixed methods as described by Creswell [10] and Creswell and Plano Clark [11]. The theory that underpins mixed methods research presumes that the collection and analysis of data consists of the combination of both qualitative and quantitative methods [11]. The design of this study was ‘sequential exploratory’, which is a two-stage design that involves using the results of the first method, the qualitative study, to build into or form the basis for the second method, the quantitative study [11].

The measures of child participation used in this study were based on previous work on children’s perspectives on school participation. An initial qualitative participative study facilitated a three-phase participative design, which actively engaged children in describing and defining what participation in school means from their own perspectives. Three schools were randomly selected from the Irish Department of Education National Primary Schools list. Workshops were organised with children aged 9–13 years in nine class groups in the three primary schools (n = 248) to gather conceptualisations and descriptions of participation in school from children’s perspective. The conceptualisations of school participation from children’s perspectives [12] formed the basis of the measurement of children’s participation in this study and were used for the development of the survey instrument. Participation in decision-making processes did not arise from the children’s conceptualisations but was added in order to more adequately represent conceptualisations of participation extant in the literature [7, 13]. This paper presents quantitative data collected by questionnaires.

The working definition of participation in school employed in this study comprised the general participation of pupils in everyday school life (including school activities and school events); decision-making by pupils; interpersonal relationships in the school environment; having a sense of belonging and ensuring equal participation of all pupils within the school.

### Participants

Participants comprised of 231 pupils aged 9–13 years in 4th, 5th and 6th classes who were recruited from nine primary schools. Three of the nine schools were designated as Health Promoting Schools (HPS) (one with only male pupils, one with only female pupils and one co-educational). The HPS were identified by the Health Promotion Department of the Health Services Executive (West) as schools that were currently actively engaging with the HPS principles, with the support of professional health promotion staff. Each HPS was matched by gender and location (i.e., urban/rural) against two NHPS in the county, which were randomly selected from the Department of Education primary school list.

### Procedure

Ethical approval for the study was granted by the National University of Ireland, Galway Research Ethics Committee. Following agreement from the principals of the schools to participate, introduction letters and information sheets were sent to schools, parents and pupils. Active consent was sought from parents and pupils. Parents were requested to return consent forms for their child to the class teacher. Only pupils whose parents gave consent, and who themselves also assented to participate by signing a consent form, completed the questionnaire.

Confidentiality of the data provided was assured and all questionnaires were anonymous. All questions were piloted before questionnaires were distributed to pupils during the school day.

### Pilot process

Three key steps were taken to pilot the survey instrument. Initially, the questionnaire was reviewed by colleagues who are working in children’s research. Their suggestions and comments on clarity and question format informed revisions to the questionnaire. The questionnaires were then piloted in a primary school with pupils from 4th, 5th and 6th class (ages 10–12 years) - a similar population to the participants in the main study. An information sheet about the study was provided to the school, teacher and pupils. Pupils were also informed that all the information from the questionnaire would be kept confidential, that taking part in the pilot was totally voluntary and that they did not have to answer any question they did not want to, or, indeed, to take part at all. The pilot questionnaires were self-administered and pupils were informed that filling in each pilot questionnaire could take about 20 minutes long, but this was one of the things that we wanted to test. A total of 27 primary school pupils participated in the pilot study.

After completing the questionnaire pupils were asked how well they understood the questionnaire, if there were any difficult words and how long it took them to complete the questionnaire (the time that each pupil returned their completed questionnaire was also recorded on their sheets). Based on feedback from the pupils, ambiguous questions were either reframed or removed and difficult words were re-worded. Third, the pilot questionnaire was again given to colleagues to validate the questions after revision. Further comments and suggestions were used to revise the questionnaire before data collection (see Additional file 1).

### Measures

Ten scales were constructed for the study and were grouped under three conceptual definitions: school participation, school socio-ecological environment (see Additional file 2) and pupil health and well-being. Negative worded items were reversed before total scores were computed for each scale and normality of scores was assessed; scale reliability was assessed to determine the scale’s internal consistency. The statistical indicator used to assess the scales’ internal consistency was the Cronbach’s alpha coefficient. Values above .6 were considered acceptable for internal consistency reliability for the scales. It has been suggested that scales with smaller number of items could have low Cronbach’s alpha coefficient values [14] as recorded in the parents’ participation in school scale. The multicollinearity of the variables was computed to show the correlations between the independent variables and the dependent variable. The tolerance value, showing how much of the variability of a stated independent variable (IV) is not explained by other independent variables in the template (that is 1-R^2^ for each variable) were higher than .10 for each scale, suggesting that the multiple correlation with other IV is low thereby signifying no possibility of multicollinearity [14]. The VIF (Variance inflation factor) values (the opposite of the Tolerance value = 1/Tolerance), were all below 10, which indicated that IV were not highly correlated.

#### School participation

School participation was measured with four scales, each assessing different dimensions of school participation; ‘participation in school decisions and rules’ comprised a six-item scale (Cronbach’s Alpha (CA) = 0.646) - one of these items (*Students take part in making school rules*) was drawn from the 2010 Health Behaviour in School-aged Children study [15, 16]; ‘Participation in school activities’ comprised eight items (CA = 0.604) (school activities were described in terms of activities that were part of every day school life, for example, arts, physical education, music, sports, drama, school tours and after school activities); ‘Participation in school events’ comprised six items (CA = 0.623) (school events were described as special events organised by schools, for example, sports day) and ‘Positive perception of school participation’ scale contained six items (CA = 0.772) (positive perception of school participation were described in terms of pupils’ perception of feeling happy about their level of participation in school).

#### Socio-ecological environment of school

Socio-ecological indicators were assessed by five scales: the intrapersonal - ‘perception of school’ (CA = 0.834); the interpersonal - ‘perceptions of class relationships’ (CA = 0.806) and ‘relationship with teacher’ (CA = 0.886); the school organisation - ‘perception of school policy’ (CA = 0.605); and the community - factors ‘parents’ participation in school life’ (CA = 0.584).

#### Pupil health and well-being

Outcome measures comprised pupils’ perceptions of their health and well-being and were measured using four questionnaire items: ‘perceived general health’, ‘self-reported happiness’, ‘self-esteem’ and ‘life satisfaction’. These items were drawn from the 2010 Health Behaviour in School-aged Children study [15, 16]. ‘Perceived general health’ and ‘self-reported happiness’ both had four response options, ‘self-esteem’ had five response options and ‘life satisfaction’ had eleven response options. The life satisfaction scale was collapsed into five groups and relabelled to ensure that life satisfaction did not have an undue influence on the overall scores. Each item was coded or recoded from low to high and then the individual scores of the four items were collapsed into a single scale with CA = 0.723.

#### Demographic characteristics

Pupils were asked to report their age, gender and class group.

### Data analysis

Associations between school participation, socio-ecological indicators, outcome measures and demographic indices were assessed using chi-square and odds ratios from logistic regression binary models. Logistic regression analysis included health and well-being outcome measures and socio-ecological dimensions of school life indicators as binary dependent variables and school participation scales as independent variables.

Analysis was conducted using SPSS version 20. Each scale item was checked for reliability to determine the scale’s internal consistency; that is, the degree to which all the items that made up each scale measured the same underlying concept. Data were screened for outliers, skewness, kurtosis and multicollinearity [14, 17, 18]. Eight cases identified as extreme outliers were removed thus reducing the total sample from 231 to 223. Total scores were computed for each scale and normality of scores was assessed.

The dependent variable scale scores were dichotomised into ‘high’ and ‘low’. The median values of the dependent variable scale scores were used as cut-off values to dichotomise into ‘high’ (the median value score and above) and ‘low’ (below the median value score). All analyses were conducted separately by gender and school category (i.e., boys and girls or HPS and NHPS). Each row in the logistic regression tables below denotes a separate logistic regression model.

## Results

### Demographic characteristics of pupils

The mean age was 10.82 years (SD 0.88). There were more (boys 54.7%; n = 122) than (girls 45.3%; n = 101) and more pupils in NHPS (64.1%; n = 143) than in designated HPS (35.9%; n = 80). Eighty pupils (35.9%) were in the 4th class, 96 (43%) in the 5th class and 47 (21.1%) in the 6th class (Table 1).

**Table 1:** **Demographic characteristic of pupils**

Demographic variable/characteristics of pupils	ALL (n = 223)	HPS (n = 80)	NHPS (n = 143)
Number (n)	n%	n%	n%
Age/age of child			
9	12 (5.4)	4 (5.0)	8 (5.6)
10	71 (31.8)	27 (33.8)	44 (30.8)
11	90 (40.4)	32 (40.0)	58 (40.6)
12	46 (20.6)	15 (18.8)	31 (21.7)
13	4 (1.8)	2 (2.5)	2 (1.4)
Gender			
Male	122 (54.7)	41(51.3)	81 (56.6)
Female	101 (45.3)	39 (48.8)	62 (43.4)
Class type			
4th	80 (35.9)	36 (45.0)	44 (30.8)
5th	96 (43.0)	31 (38.8)	65 (45.5)
6th	47 (21.1)	13 (16.3)	34 (23.8)

Note: Health Promoting School (HPS); Non-Health Promoting School (NHPS).

### Extent of pupil participation in school

Overall, the mean score for participation in school decisions and rules was 15.81 (SD 3.57); the mean score for participation in school activities was 22.25 (SD 3.42); the mean score for participation in school events was 17.08 (SD 3.61); and the mean score for reported positive perception of school participation was 26.20 (SD 3.36). The differences in the reported mean score for participation in school decisions and rules, school activities and school events scales among pupils in HPS and NHPS were marginal and not statistically significant. However, the mean score for reported positive perception of school participation was significantly lower (χ^2^ = 5.13, df =1, p < 0.05) among pupils in HPS (mean = 26.03; SD 3.37) compared to NHPS (mean = 26.30; SD 3.36) (Table 2). The mean score was significantly higher for participation in school decisions and rules among boys in HPS (χ^2^ = 7.06, df =1, p < 0.01) compared to NHPS but significantly lower among girls in HPS across all school participation indicators apart from participation in school activities in which differences were not statistically significant (see Table 2).

**Table 2:** **Mean school participation scores, overall and by gender and school category**

	Participation in school (Mean scores and Standard Deviations)			
	Participation in school decisions and rules	Participation in school activities	Participation in school events	Positive perception of school participation
	Range 6-30	Range 10-30	Range 6-30	Range 10-35
	Mean (SD)	Mean (SD)	Mean (SD)	Mean (SD)
All (n = 223)	15.81 (3.57)	22.25(3.42)	17.08 (3.61)	26.20 (3.36)
All HPS^1^ (n = 80)	15.89 (3.99)	22.18(3.22)	16.78( 3.60)	26.03 (3.37)
All NHPS^2^ (n = 143)	15.77 (3.32)	22.29(3.54)	17.25 (3.62)	26.30 (3.36)
	χ^2^ = 0.09, df = 1, p > 0.05	χ^2^ = 0.06, df = 1, p > 0.05	χ^2^ = 0.00, df = 1, p > 0.05	χ^2^ = 5.13, df = 1, p < 0.05
All boys (n = 122)	16.19 (3.30)	21.82 (3.25)	16.69(3.22)	25.77 (3.67)
HPS boys (n = 41)	17.65 (2.98)	23.17 (2.92)	17.78(3.55)	26.68 (3.64)
NHPS boys (n = 81)	15.46 (3.23)	21.14 (3.21)	16.14(2.91)	25.31 (3.62)
Chi square	χ^2^ = 7.06, df = 1,	χ^2^ = 1.42, df = 1,	χ^2^ = 3.54, df = 1,	χ^2^ = 0.40, df = 1,
	p < 0.01	p = 0.23	p = 0.06	p = 0.53
All girls (n = 101)	15.35 (3.83)	22.76 (3.57)	17.55(4.0)	26.72 (2.88)
HPS girls (n = 39)	14.08 (4.13)	21.13 (3.23)	15.72(3.38)	25.36 (2.96)
NHPS girls (n = 62)	16.18 (3.42)	23.79 (3.40)	18.71(3.95)	27.59 (2.48)
Chi square	χ^2^ = 5.60, df = 1, p < 0.05	χ^2^ = 2.35, df = 1, p = 0.13	χ^2^ = 4.03, df = 1, p < 0.05	χ^2^ = 18.83, df = 1, p < 0.001

^1^Health Promoting Schools (HPS); ^2^Non-Health Promoting Schools (NHPS).

### School participation and health and well-being of pupils

Overall, the mean score for the health and well-being outcome measure was 15.26 (SD 2.44). The mean scores were similar for HPS (15.00, SD 2.61) and NHPS (15.40, SD 2.33); this difference was not statistically significant. As shown in Table 3, the univariate logistic regression analyses revealed that school participation indicators were significantly associated with reported positive health and well-being. Participation in school decisions and rules (OR 1.22, 95% CI 1.12-1.33); participating in school activities (OR 1.20, 95% CI 1.10-1.31); participating in school events (OR 1.19, 95% CI 1.10-1.29) and reported positive perception of school participation (OR 1.26, 95% CI 1.15-1.39) were all positively associated with health and well-being outcomes for all pupils, and for each sub-group.

**Table 3:** **Relative odds of self-rated health and well-being outcomes associated with participation in school, overall and by gender and school category**

***Univariate analyses***	Health and well-being				
	All	HPS ^1^	NHPS ^2^	Boys	Girls
Participation in school decisions and rules	1.22***	1.32***	1.16**	1.16*	1.28***
	(1.12-1.33)	(1.14-1.53)	(1.04-1.29)	(1.03-1.31)	(1.13-1.46)
Participation in school activities	1.20***	1.23**	1.19**	1.23**	1.21**
	(1.10-1.31)	(1.05-1.43)	(1.07-1.32)	(1.08-1.40)	(1.06-1.37)
Participation in school events	1.19***	1.26**	1.16**	1.25**	1.17**
	(1.10-1.29)	(1.09-1.46)	(1.05-1.28	(1.10-1.42)	(1.05-1.30)
Reported positive perception of school participation	1.26***	1.31**	1.23***	1.24***	1.33**
	(1.15-1.39)	(1.11-1.53)	(1.10-1.38)	(1.11-1.40)	(1.13-1.57)
***Multivariate analyses***	**All**	**HPS** ^**1**^	**NHPS** ^**2**^	**Boys**	**Girls**
Participation in school decisions and rules	1.10	1.20*	1.04	0.99	1.19*
	(1.0-1.22)	(1.01-1.42)	(0.91-1.19)	(0.86-1.15)	(1.02-1.39)
Participation in school activities	1.04	1.00	1.08	1.10	1.05
	(0.92-1.18)	(0.82-1.23)	(0.92-1.27)	(0.92-1.31)	(0.86-1.28)
Participation in school events	1.03	1.09	1.00	1.10	0.96
	(0.92-1.16)	(0.89-1.33)	(0.87-1.15	(0.93-1.30)	(0.80-1.16)
Reported positive perception of school participation	1.20**	1.16	1.20*	1.18*	1.22
	(1.06-1.35)	(0.94-1.42)	(1.03-1.39)	(1.00-1.38)	(1.00-1.50)

Notes: *p < 0.05; **p < 0.01; ***p < 0.001.

^1^Health Promoting Schools (HPS); ^2^Non-Health Promoting Schools (NHPS).

When all the school participation scales were entered together, with health and well-being as the dependent variable, participation in school decisions and rules, participating in school activities, participating in school events and reported positive perception of school participation as predictor variables, a total of 215 cases were analysed. Multivariate analyses showed that the full model containing all predictors was statistically significant, χ^2^ (4, N = 215) = 39.98, P < 0.001; these results indicate that the model was able to distinguish between pupils who reported and did not report positive health and well-being outcomes. The model as a whole correctly classified 67.9% of cases. Table 3 shows that only reported positive perception of school participation (OR 1.20, 95% CI 1.06-1.35) was positively associated with health and well-being outcomes for all pupils; none of the other school participation predictors were significantly associated with reported health and well-being outcomes. However, pupils in NHPS (OR 1.20, 95% CI 1.03-1.39) and boys (OR 1.18, 95% CI 1.00-1.38) were more likely to report positive health and well-being outcomes for reported positive perception of school participation than pupils in HPS and girls. On the other hand, pupils in HPS (OR 1.20, 95% CI 1.01-1.42) and girls (OR 1.19, 95% CI 1.02-1.39) were more likely to report positive health and well-being outcomes for participation in school decisions and rules than pupils in NHPS and boys (see Table 3).

### School participation and socio-ecological indicators

As shown in Table 4, the simple logistic regression analyses revealed that there were positive associations between each school participation scale and socio-ecological relationships at school. Participation in school decisions and rules (OR 1.58, 95% CI 1.29-1.93) was positively associated with reported positive perception of school in HPS (Table 4). Conversely, participating in school activities (OR 1.63, 95% CI 1.39-1.92) and school events (OR 1.48, 95% CI 1.29-1.70) were more likely to be significantly associated with perceptions of school policies in NHPS while reported positive perception of school participation was more likely to be significantly associated with class relationships (OR 1.71, 95% CI 1.41-2.07) and relationship with teacher (OR 1.61, 95% CI 1.35-1.93) in NHPS (Table 4).

**Table 4:** **Relative odds of school socio-ecological environment associated with school participation, overall and by gender and school category**

School participation	School socio-ecological environment				
	Perception of school				
	All	HPS ^1^	NHPS ^2^	Boys	Girls
Participation in school decisions and rules	1.25***	1.58***	1.12*	1.26**	1.29***
	(1.15-1.37)	(1.29-1.93)	(1.01-1.25)	(1.11-1.43)	(1.13-1.47)
Participation in school activities	1.34***	1.23**	1.40***	1.56***	1.19**
	(1.21-1.48)	(1.06-1.44)	(1.23-1.60)	(1.31-1.85)	(1.05-1.34)
Participation in school events	1.24***	1.31**	1.21***	1.30***	1.19**
	(1.14-1.35)	(1.12-1.54)	(1.09-1.34)	(1.13-1.49)	(1.06-1.34)
Reported positive perception of school participation	1.43***	1.53***	1.37***	1.40***	1.45***
	(1.28-1.59)	(1.26-1.85)	(1.20-1.57)	(1.21-1.62)	(1.22-1.74)
	**Class relationships**				
	**All**	**HPS** ^**1**^	**NHPS** ^**2**^	**Boys**	**Girls**
Participation in school decisions and rules	1.30***	1.23**	1.36***	1.32***	1.30***
	(1.18-1.42)	(1.08-1.40)	(1.19-1.55)	(1.15-1.52)	(1.14-1.48)
Participation in school activities	1.43***	1.30**	1.52***	1.63***	1.29***
	(1.28-1.59)	(1.10-1.53)	(1.31-1.77)	(1.35-1.97)	(1.13-1.48)
Participation in school events	1.24***	1.15*	1.31***	1.26**	1.23**
	(1.14-1.36)	(1.01-1.31)	(1.16-1.48)	(1.10-1.44)	(1.09-1.38)
Reported positive perception of school participation	1.49***	1.31**	1.71***	1.57***	1.41***
	(1.32-1.69)	(1.12-1.54)	(1.41-2.07)	(1.32-1.87)	(1.18-1.68)
	**Relationship with teacher**				
	**All**	**HPS** ^1^	**NHPS** ^2^	**Boys**	**Girls**
Participation in school decisions and rules	1.24***	1.22**	1.26***	1.26**	1.26**
	(1.13-1.36)	(1.07-1.40)	(1.11-1.42)	(1.10-1.44)	(1.10-1.43)
Participation in school activities	1.44***	1.37***	1.49***	1.62***	1.32***
	(1.29-1.62)	(1.15-1.64)	(1.29-1.73)	(1.34-1.97)	(1.15-1.53)
Participation in school events	1.26***	1.28**	1.25***	1.24* *	1.28** *
	(1.15-1.38)	(1.10-1.50)	(1.11-1.40)	(1.09-1.42)	(1.12-1.45)
Reported positive perception of school participation	1.57***	1.51***	1.61***	1.49***	1.72***
	(1.38-1.79)	(1.25-1.83)	(1.35-1.93)	(1.28-1.74)	(1.38-2.15)
	**Perceptions of school policies**				
	**All**	**HPS** ^**1**^	**NHPS** ^**2**^	**Boys**	**Girls**
Participation in school decisions and rules	1.22***	1.22**	1.22**	1.26**	1.26***
	(1.12-1.33)	(1.07-1.38)	(1.09-1.37)	(1.10-1.43)	(1.11-1.43)
Participation in school activities	1.60***	1.55***	1.63***	1.56***	1.61***
	(1.41-1.82)	(1.26-1.91)	(1.39-1.92)	(1.31-1.85)	(1.33-1.94)
Participation in school events	1.44***	1.39***	1.48***	1.33***	1.60***
	(1.29-1.61)	(1.17-1.65)	(1.29-1.70)	(1.16-1.53)	(1.33-1.92)
Reported positive perception of school participation	1.47***	1.39***	1.54***	1.44***	1.51***
	(1.30-1.65)	(1.17-1.65)	(1.30-1.82)	(1.23-1.69)	(1.25-1.81)
	**Parents’ participation in school life**				
	**All**	**HPS** ^**1**^	**NHPS** ^**2**^	**Boys**	**Girls**
Participation in school decisions and rules	1.22***	1.24**	1.21**	1.15*	1.33***
	(1.12-1.33)	(1.08-1.42)	(1.08-1.36)	(1.02-1.30)	(1.16-1.52)
Participation in school activities	1.22***	1.16 ^**+**^	1.25***	1.14*	1.30***
	(1.11-1.33)	(1.0-1.34)	(1.12-1.39)	(1.02-1.29)	(1.13-1.49)
Participation in school events	1.22***	1.14^**++**^	1.27***	1.18**	1.25***
	(1.12-1.33)	(1.0-1.31)	(1.13-1.42)	(1.04-1.34)	(1.11-1.42)
Reported positive perception of school participation	1.27***	1.25**	1.27***	1.20**	1.39***
	(1.15-1.39)	(1.07-1.46)	(1.13-1.43)	(1.07-1.34)	(1.18-1.65)

Notes: *p < 0.05; **p < 0.01; ***p < 0.001; ^+^p = 0.056; ^++^p = 0.05.

^1^Health Promoting Schools (HPS); ^2^Non-Health Promoting Schools (NHPS).

For girls, reported positive perception of school participation (OR 1.72, 95% CI 1.38-2.15) was positively associated with relationship with teacher while participation in school activities (OR 1.61, 95% CI 1.33-1.94) and school events (OR 1.60, 95% CI 1.33-1.92) was positively associated with perceptions of school policies (see Table 4). For boys participation in school activities was more likely to be significantly associated with class relationships (OR 1.63, 95% CI 1.35-1.97) and relationship with teacher (OR 1.62, 95% CI 1.34-1.97) (see Table 4).

## Discussion

This study expands on existing research on child participation in school life; the dimensions of school participation used as measures of child participation in this study were based on the perspectives of children themselves, thus indicating a broader and more externally valid definition of participation than any previous work. This study suggests that when considering measurements related to the concepts of school participation, it is important to consider the views of children and so, while developing indices, children’s participation in school might encompass more dimensions than previously outlined in the literature.

The findings from this study identified that, overall, pupils had a relatively positive perception of participating in school life. Results indicate that the more pupils participate in everyday school life, the more likely they are to report positive health and well-being outcomes. A notable finding from this study is that it empirically links school participation with health and well-being; this had only been previously documented by de Roiste et al., [9]. However, this study is different in that it highlights school participation among younger children (in primary schools) with a much broader definition of school participation, and an explicit look at differences and/or similarities in HPS and NHPS schools.

The mean scores for other dimensions of school participation were marginal between HPS and NHPS except for positive perception of school participation. It might be expected that schools that were reportedly actively engaging with the HPS principles would demonstrate higher levels of pupil participation. However being a HPS school in this context means a school that is striving to become ‘Health Promoting’, and thus is working on the application of the core concepts and principles of HPS – but may not yet have achieved them from any objective perspective. In addition, as the principles of HPS are derived in part from good educational practice, it may well be that schools categorised as NHPS are engaged in similar activities.

Participation in school has previously been linked to the health and well-being of pupils [9]. Findings from the current study corroborate this and have identified that all dimensions of school participation were positively associated with reported positive health and well-being outcomes, and the multivariate model provides an indication of the unique contribution of pupils’ positive perception of school participation above that of other measures of school participation. This finding implies that pupils’ health and well-being could be improved by encouraging improvements in the participation of children in school, albeit while also attending to other factors that affect pupils’ health and well-being within the school setting. It has been suggested that school participation has the potential to enhance positive health outcomes and improve pupils’ perception of their school [9]. School participation and health and well-being outcomes are not systematically different between HPS and NHPS, though there are some marginal differences. The HPS seeks to improve the health of pupils and the whole school environment and it has been documented that pupils in schools that are endeavouring to implement the whole school approach within the context of the HPS concept show more positive health improvements than those in schools that are not yet doing so [19]. The non-systematic differences between HPS and NHPS in this study therefore suggest that there needs to be better clarity between factors that distinguish HPS from NHPS among Irish schools.

These data also show that school participation was positively associated with the socio-ecological school environment across school categories. It has been previously suggested that the close connection or association of children with their environment, for example schools, has the potential to act as a strong determinant in enhancing children’s participation in the school setting [5]. The school environment is considered an important component of the HPS approach [6, 7]. For example, student participation has been linked with improved student-adult relationships [20]. Griebler and Nowak also showed some effects of student participation on student-adult relationships but these were reported mostly among pupils involved in the student councils [13]. Children’s relationships with their teachers and other adults in their lives and immediate environment have the potential to positively influence pupil participation in school and to influence children’s lives [5].

School participation has the potential to enhance the socio-ecological school environment and school relationships. The link between school participation and socio-ecological factors in schools, as shown in this study, has been previously demonstrated in specific intervention projects where the participation focused on pupil involvement in the intervention. However, this study identified a positive association from a more general perspective on participation of children in everyday school life.

### Strengths and limitations of the study

The concept of participation (participation in school activities, school events, school decisions and rules and positive perception of school participation) measured in this study was based on children’s descriptions of what participation in school meant to them and indicates that school participation is potentially valuable for improved school outcomes and to enhance socio-ecological relationships within the school.

However, every school goes through a developmental process, improvements are not static and there could be shifts over time. This study only shows one perspective on school participation, and highlights some differences between HPS and NHPS, but suggests that school participation is important for children e.g., [21]. The lack of systematic differences between HPS and NHPS could be due to the methods of the study, or the categorisation of the schools; but it is clear that further qualitative research is needed to identify essential characteristics that define a HPS in the Irish context. In addition there are likely to be a range of other, unmeasured, factors that influence all the concepts included here, and particularly the health and well-being outcomes.

Furthermore, data from this study were limited to nine primary schools and to pupils in the 4th, 5th and 6th classes. A more heterogeneous group and a larger study sample would be required for greater representativeness. This study employed a cross-sectional design and causal relationships cannot be implied, neither can the direction of any documented relationships.

## Conclusions

The findings from this study indicate that there is a relationship between school participation and positive health and well-being of pupils and socio-ecological school relationships. This study highlights that school participation is important for children in school, is related to health and well-being outcomes and could enhance positive socio-ecological relationships within the school, although the direction and nature of these relationships requires more study. In addition, the relationships between these indicators highlight a potential justification for encouraging the development of this HPS concept within schools. Interventions in promoting participation at the school level and effects on child outcomes could be targeted to the school socio-ecological environment. School policies that encourage more engagement with school activities and events could be promoted.

## Authors’ information

Yetunde John-Akinola, B.Sc., MPH, is a PhD student in the Health Promotion Department of the School of Health Sciences, National University of Ireland, Galway. She is a lecturer at the Department of Health Promotion and Education, University of Ibadan Nigeria. Her research interests include school health promotion and health promoting schools, children’s participation in school and in the research process, and adolescent and young people’s health. Saoirse Nic Gabhainn, Ph.D., is Senior Lecturer in Health Promotion, School of Health Sciences, National University of Ireland, Galway. Her research interests include child health and well-being, adolescent risk behaviour, health promoting schools and child participation in the research process.

## Electronic supplementary material

Additional file 1: **The survey questionnaire for the study.** (DOCX 81 KB)

Additional file 2: **School participation and school socio-ecological environment scales.** (DOCX 18 KB)

## Abbreviations

HPS: Health Promoting Schools
NHPS: Non-Health Promoting Schools.
