# Supplementary material for: Children’s participation in school: a cross-sectional study of the relationship between school environments, participation and health and well-being outcomes
Source: BMC Public Health. 2014 Sep 17;14:964. doi: 10.1186/1471-2458-14-964 (PMC4177162; doi:10.1186/1471-2458-14-964)
Supplement: Supplementary file 1 — Additional file 1: The survey questionnaire for the study. (DOCX 81 KB) [file 12889_2014_7073_MOESM1_ESM.docx]

**ADDITIONAL FILE 1**

**THE SURVEY QUESTIONNAIRE FOR THE STUDY**

**TITLE: Children's participation in school: a cross-sectional study of the relationship between school environments, participation and health and well-being outcomes**


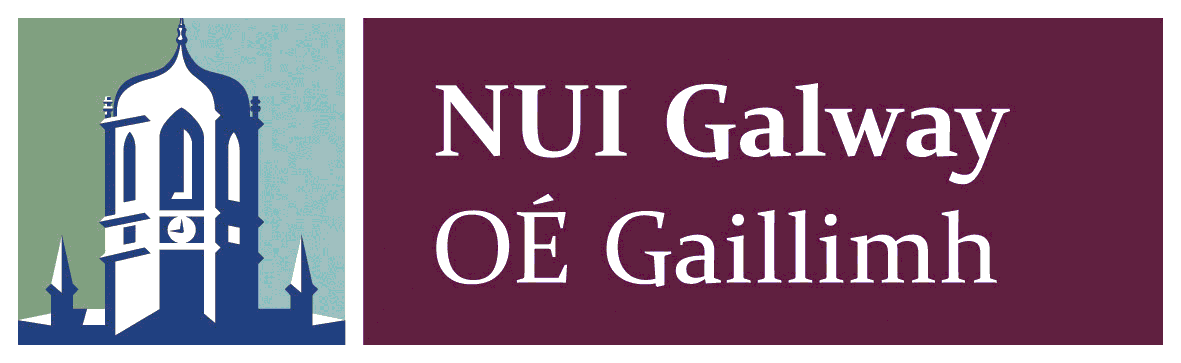


**
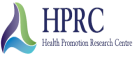
**

**Children's participation in school: a cross-sectional study of the relationship between school environments, participation and health and wellbeing outcomes**

**Introduction**

The questions in this questionnaire are about what other children in primary schools, like you, have told me. It is about how you take part (participate) in your school and how taking part in your school can help you. I would like you to answer the questions because your answers will be very useful to find out how children in primary schools take part (participate) in their schools. Other children in primary schools in Galway County will also be filling in this questionnaire.

**Please note:**

- You do not need to put your name on the questionnaire
- Please read each question carefully before you answer and answer it as honestly as you can
- There is no right or wrong answer. What you think is very important to this research study
- You are free to take part or not take part in the study. It is your choice
- You do not have to answer any question you do not want to
- You will be asked to tick the box that best fits your answer or write on the lines

Thank you for your time.

**Example:**

**1. Is Galway in the west of Ireland?**

**Yes No**

🗸

If you find it hard to tick just one answer, please select the answer that you think is **true most of the time**.

**1.**

1. **ABOUT YOU**

| **Are you a boy or a girl?** |  |
| --- | --- |
| **Boy** □ | **Girl** □ |

| **How old are you?** | | | | | |
| --- | --- | --- | --- | --- | --- |
| □ | □ | □ | □ | □ | □ |
| **9 years** | **10 years** | **11 years** | **12 years** | **13 years** | **14 years** |

| **What class are you in?** | | |
| --- | --- | --- |
| □ | □ | □ |
| **4^th^ class** | **5^th^ class** | **6^th^ class** |

**2. ABOUT YOUR SCHOOL**

Here are some sentences about **your school**. Please tick a box to show how you feel about each sentence. *Please tick only one box for each sentence*.

|  | **Please tick 🗸** **one box for each sentence** | Always | Often | Sometimes | Never |
| --- | --- | --- | --- | --- | --- |
| 1 | Are you happy going to your school every day? | □ | □ | □ | □ |
| 2 | Do you look forward to going to your school? | □ | □ | □ | □ |
| 3 | I like my school | □ | □ | □ | □ |
| 4 | I feel I am a part of my school | □ | □ | □ | □ |
| 5 | I am happy to be a part of this school | □ | □ | □ | □ |
| 6 | Students’ needs are important in our school | □ | □ | □ | □ |
| 7 | I feel comfortable in my school | □ | □ | □ | □ |
| 8 | In our school I feel safe | □ | □ | □ | □ |
| 1. **ABOUT YOUR SCHOOL POLICY** | | | | | |
|  | **Please tick 🗸** **one box for each sentence** | Always | Often | Sometimes | Never |
| 1 | In our school, students work together to design or plan their own school activity/school event | □ | □ | □ | □ |
| 2 | In our school, we do activities that everyone enjoys | □ | □ | □ | □ |
| 3 | In our school, everybody is included in fun and games | □ | □ | □ | □ |
| 4 | In our school students are allowed to pick on other students | □ | □ | □ | □ |
| 5 | In our school students are made to do something even if they don’t want to do it | □ | □ | □ | □ |
| 6 | Our school is a fun place to be | □ | □ | □ | □ |

**4. ABOUT SCHOOL ACTIVITIES**

| 1. What type of activities do you do in school? **Please tick 🗸** **all activities you do** | | | | | | | | | | |  |  |  |
| --- | --- | --- | --- | --- | --- | --- | --- | --- | --- | --- | --- | --- | --- |
| □ | □ | □ | □ | □ | | □ | | | □ | |  |  |  |
| Sports | Arts | Music | Drama | Physical Education (PE) | | School Tours | | | After school activities | |  |  |  |
| Other (please write it down): _____________________________________________ | | | | | | | | | | |  |  |  |
| **For the activities you ticked above** | | | | | | | Always | | Often | Sometimes | | Never | |
| 2 | How often do you take part in the activities above? | | | | | | □ | | □ | □ | | □ | |
| 3 | Do you have fun doing the activities above? | | | | | | □ | | □ | □ | | □ | |
| 4 | Do you feel you spend enough time doing the activities above? | | | | | | □ | | □ | □ | | □ | |
| 5 | Students’ views are important in planning school activities | | | | | | □ | | □ | □ | | □ | |
| 6 | In our school, students are allowed to take part in any school activity they are interested in | | | | | | □ | | □ | □ | | □ | |
| 7 | Students can say if school activities they took part in were good or not | | | | | | □ | | □ | □ | | □ | |
| 8 | I enjoy doing school activities with other students | | | | | | □ | | □ | □ | | □ | |
| 1. **ABOUT SCHOOL EVENTS Please tick** 🗸 **one box for each sentence** | | | | | | | | | | | | | |
| 1 | Students take part in planning school events | | | | | | □ | | □ | □ | | □ | |
| 2 | Students are sure about how to take part in school events | | | | | | □ | | □ | □ | | □ | |
| 3 | Students are told about how important their taking part was to the success of school events | | | | | | □ | | □ | □ | | □ | |
| 4 | Students can stop taking part in any school event if they want to | | | | | | □ | | □ | □ | | □ | |
| 5 | Students are told how to take part in school events | | | | | | □ | | □ | □ | | □ | |
| 6 | In our school, students take action to see if school events they took part in were okay | | | | | | □ | | □ | □ | | □ | |

1. **ABOUT SCHOOL DECISIONS AND RULES**

Here are some sentences about **school decisions and rules**. Please tick a box to show how best you feel about each sentence. *Please tick only one box for each sentence*.

|  | **Please tick 🗸one box for each sentence** | Always | Often | Sometimes | Never |
| --- | --- | --- | --- | --- | --- |
| 1 | Students take part in making school rules | □ | □ | □ | □ |
| 2 | Students’ views are listened to in our school | □ | □ | □ | □ |
| 3 | In our school, students are allowed to have a say in what concerns them | □ | □ | □ | □ |
| 4 | Students in our school are allowed to say how they feel | □ | □ | □ | □ |
| 5 | In our school, students’ views are acted on | □ | □ | □ | □ |
| 6 | In our school, we have students who speak for other students | □ | □ | □ | □ |

1. **ABOUT YOUR CLASS**

Here are some sentences about **your class**. Please show how much you agree or disagree with each sentence. *Please tick only one box for each sentence*.

|  | **Please tick 🗸one box for each sentence** | Strongly agree | Agree | Neither agree nor disagree | Disagree | Strongly disagree |
| --- | --- | --- | --- | --- | --- | --- |
| 1 | My friends are important in making me feel a part of my class | □ | □ | □ | □ | □ |
| 2 | Students in my class are encouraged to write down their ideas about things they are interested in doing in the school | □ | □ | □ | □ | □ |
| 3 | Students in my class support each other and this makes me feel comfortable | □ | □ | □ | □ | □ |
| 4 | Students in my class help each other and this makes me feel a part of my class | □ | □ | □ | □ | □ |
| 5 | Students in my class enjoy working together on projects | □ | □ | □ | □ | □ |
| 6 | I like reading together with other students in my class and this makes me feel a part of my class | □ | □ | □ | □ | □ |
| 7 | In my class it is important not to leave anybody out | □ | □ | □ | □ | □ |
| 8 | Our classroom is a nice place for learning | □ | □ | □ | □ | □ |

1. **ABOUT TAKING PART IN SCHOOL**

Here are some sentences about **taking part in your school**. Please show how much you agree or disagree with each sentence. *Please tick only one box for each sentence*.

|  | **Please tick 🗸one box for each sentence** | Strongly agree | Agree | Neither agree nor disagree | Disagree | Strongly disagree |
| --- | --- | --- | --- | --- | --- | --- |
| 1 | All students have the right to take part in our school | □ | □ | □ | □ | □ |
| 2 | Helping students to take part has made our school more lively | □ | □ | □ | □ | □ |
| 3 | Giving students rewards on all they do can encourage participation in our school | □ | □ | □ | □ | □ |
| 4 | Taking part in school is fun | □ | □ | □ | □ | □ |
| 5 | Participation in school activities makes me feel healthy | □ | □ | □ | □ | □ |
| 6 | I feel happy about my level of participation in my school | □ | □ | □ | □ | □ |

1. **ABOUT YOUR TEACHER(S)**

Here are some sentences about **your teacher(s)**. Please show how much you agree or disagree with each sentence. *Please tick only one box for each sentence*.

|  | **Please tick 🗸one box for each sentence** | Strongly agree | Agree | Neither agree nor disagree | Disagree | Strongly disagree |
| --- | --- | --- | --- | --- | --- | --- |
| 1 | My teacher(s) make me feel a part of my school | □ | □ | □ | □ | □ |
| 2 | My teacher(s) make me feel comfortable | □ | □ | □ | □ | □ |
| 3 | Our teacher(s) encourage us to say what we think in the class | □ | □ | □ | □ | □ |
| 4 | Our teacher(s) are nice | □ | □ | □ | □ | □ |
| 5 | I like my teacher(s) | □ | □ | □ | □ | □ |

**10.** **ABOUT YOU, YOUR HEALTH AND HOW YOU FEEL**

**Please tick 🗸one box for each sentence**

| **1. Would you say your health is.....?** | | | |
| --- | --- | --- | --- |
| **Excellent** □ | **Good** □ | **Fair** □ | **Poor** □ |
| **2. In general how do you feel about your life at present?** | | | |
| **I feel very happy** □ | **I feel quite happy** □ | **I don’t feel very happy** □ | **I’m not happy at all** □ |

| **3. Thinking about the last week.....**  **Have you been happy with the way you are?** | | | | |
| --- | --- | --- | --- | --- |
| **Never** □ | **Seldom** □ | **Quite often** □ | **Very often** □ | **Always** □ |

**4. Here is a picture of a ladder:**

The top of the ladder ‘10’ is the best possible life for you and the bottom ‘0’ is the worst possible life for you.

**In general, where on the ladder do you feel you stand at the moment?**

**Please tick 🗸next to the number that best describes where you stand.**

|  |  |  |
| --- | --- | --- |
|  | **10** | **Best possible life** |
|  | **9** |  |
|  | **8** |  |
|  | **7** |  |
|  | **6** |  |
|  | **5** |  |
|  | **4** |  |
|  | **3** |  |
|  | **2** |  |
|  | **1** |  |
|  | **0** | **Worst possible life** |
|  |  |  |

**13. ABOUT YOUR PARENTS**

Here are some sentences about **your parents**. Please tick one box to show how best you feel about each sentence. *Please tick one box for each sentence*.

|  | **Please tick 🗸one box for each sentence** | Always | Often | Sometimes | Never |
| --- | --- | --- | --- | --- | --- |
| 1 | My parents are involved in our school activities | □ | □ | □ | □ |
| 2 | My parents are made to feel a part of our school | □ | □ | □ | □ |
| 3 | My parents are encouraged to talk about things that concern me in our school | □ | □ | □ | □ |

**Thank you for taking the time to fill in this questionnaire**
