# Supplementary material for: Children’s participation in school: a cross-sectional study of the relationship between school environments, participation and health and well-being outcomes
Source: BMC Public Health. 2014 Sep 17;14:964. doi: 10.1186/1471-2458-14-964 (PMC4177162; doi:10.1186/1471-2458-14-964)
Supplement: Supplementary file 2 — Additional file 2: School participation and school socio-ecological environment scales. (DOCX 18 KB) [file 12889_2014_7073_MOESM2_ESM.docx]

**ADDITIONAL FILE 2**

**School participation and school socio-ecological environment scales**

1. School participation scales

| **Participation in school decisions and rules** | | Always | Often | Sometimes | Never |
| --- | --- | --- | --- | --- | --- |
| 1. Students take part in making school rules | | □ | □ | □ | □ |
| 2. Students’ views are listened to in our school | | □ | □ | □ | □ |
| 3. In our school, students are allowed to have a say in what concerns them | | □ | □ | □ | □ |
| 4. Students in our school are allowed to say how they feel | | □ | □ | □ | □ |
| 5. In our school, students’ views are acted on | | □ | □ | □ | □ |
| 6. In our school, we have students who speak for other students | | □ | □ | □ | □ |
| Participation in school activities (Arts, physical education, music, sports, drama, school tours and after school activities) | | Always | Often | Sometimes | Never |
| 1. How often do you take part in the activities above? | | □ | □ | □ | □ |
| 2. Do you have fun doing the activities above? | | □ | □ | □ | □ |
| 3. Do you feel you spend enough time doing the activities above? | | □ | □ | □ | □ |
| 4. Students’ views are important in planning school activities | | □ | □ | □ | □ |
| 5. In our school, students are allowed to take part in any school activity they are interested in | | □ | □ | □ | □ |
| 6. Students can say if school activities they took part in were good or not | | □ | □ | □ | □ |
| 7. I enjoy doing school activities with other students | | □ | □ | □ | □ |
| Participation in school events | |  |  |  |  |
| 1. Students take part in planning school events | | □ | □ | □ | □ |
| 2. Students are sure about how to take part in school events | | □ | □ | □ | □ |
| 3. Students are told about how important their taking part was to the success of school events | | □ | □ | □ | □ |
| 4. Students can stop taking part in any school event if they want to | | □ | □ | □ | □ |
| 5. Students are told how to take part in school events | | □ | □ | □ | □ |
| 6. In our school, students take action to see if school events they took part in were okay | | □ | □ | □ | □ |
| Positive perception of school participation | Strongly agree | Agree | Neither agree nor disagree | Disagree | Strongly disagree |
| 1. All students have the right to take part in our school | □ | □ | □ | □ | □ |
| 2. Helping students to take part has made our school more lively | □ | □ | □ | □ | □ |
| 3. Giving students rewards on all they do can encourage participation in our school | □ | □ | □ | □ | □ |
| 4. Taking part in school is fun | □ | □ | □ | □ | □ |
| 5. Participation in school activities makes me feel healthy | □ | □ | □ | □ | □ |
| 6. I feel happy about my level of participation in my school | □ | □ | □ | □ | □ |

2. Socio-ecological school environment

| Perception of school | | | Always | Often | Sometimes | Never |
| --- | --- | --- | --- | --- | --- | --- |
| 1 | Are you happy going to your school every day? | | □ | □ | □ | □ |
| 2 | Do you look forward to going to your school? | | □ | □ | □ | □ |
| 3 | I like my school | | □ | □ | □ | □ |
| 4 | I feel I am a part of my school | | □ | □ | □ | □ |
| 5 | I am happy to be a part of this school | | □ | □ | □ | □ |
| 6 | Students’ needs are important in our school | | □ | □ | □ | □ |
| 7 | I feel comfortable in my school | | □ | □ | □ | □ |
| 8 | In our school I feel safe | | □ | □ | □ | □ |
|  | Perception of school policy | | Always | Often | Sometimes | Never |
| 1 | In our school, students work together to design or plan their own school activity/school event | | □ | □ | □ | □ |
| 2 | In our school, we do activities that everyone enjoys | | □ | □ | □ | □ |
| 3 | In our school, everybody is included in fun and games | | □ | □ | □ | □ |
| 4 | In our school students are allowed to pick on other students | | □ | □ | □ | □ |
| 5 | In our school students are made to do something even if they don’t want to do it | | □ | □ | □ | □ |
| 6 | Our school is a fun place to be | | □ | □ | □ | □ |
|  | Perception of class relationships | Strongly agree | Agree | Neither agree nor disagree | Disagree | Strongly disagree |
| 1 | My friends are important in making me feel a part of my class | □ | □ | □ | □ | □ |
| 2 | Students in my class are encouraged to write down their ideas about things they are interested in doing in the school | □ | □ | □ | □ | □ |
| 3 | Students in my class support each other and this makes me feel comfortable | □ | □ | □ | □ | □ |
| 4 | Students in my class help each other and this makes me feel a part of my class | □ | □ | □ | □ | □ |
| 5 | Students in my class enjoy working together on projects | □ | □ | □ | □ | □ |
| 6 | I like reading together with other students in my class and this makes me feel a part of my class | □ | □ | □ | □ | □ |
| 7 | In my class it is important not to leave anybody out | □ | □ | □ | □ | □ |
| 8 | Our classroom is a nice place for learning | □ | □ | □ | □ | □ |
|  | Relationship with teacher | Strongly agree | Agree | Neither agree nor disagree | Disagree | Strongly disagree |
| 1 | My teacher(s) make me feel a part of my school | □ | □ | □ | □ | □ |
| 2 | My teacher(s) make me feel comfortable | □ | □ | □ | □ | □ |
| 3 | Our teacher(s) encourage us to say what we think in the class | □ | □ | □ | □ | □ |
| 4 | Our teacher(s) are nice | □ | □ | □ | □ | □ |
| 5 | I like my teacher(s) | □ | □ | □ | □ | □ |
|  | Parents participation in school | Always | Often | Sometimes | Never | |
| 1 | My parents are involved in our school activities | □ | □ | □ | □ | |
| 2 | My parents are made to feel a part of our school | □ | □ | □ | □ | |
| 3 | My parents are encouraged to talk about things that concern me in our school | □ | □ | □ | □ | |
